# The Long-Term Outcome and Quality of Life after Replacement of the Ascending Aorta †

**DOI:** 10.3390/jcm12134498

**Published:** 2023-07-05

**Authors:** Marwan Hamiko, Katja Jahnel, Julia Rogaczewski, Myriam Schafigh, Miriam Silaschi, Andre Spaeth, Markus Velten, Wilhelm Roell, Ali El-Sayed Ahmad, Farhad Bakhtiary

**Affiliations:** 1 Department of Cardiac Surgery, University Hospital Bonn, Venusberg Campus 1, 53127 Bonn, Germany; katja.jahnel@gmx.de (K.J.); julia.rogaczewski@gmail.de (J.R.); myriam.schafigh@ukbonn.de (M.S.); miriam.silaschi@ukbonn.de (M.S.); andre.spaeth@ukbonn.de (A.S.); wilhelm.roell@ukbonn.de (W.R.); ali.assayed@googlemail.de (A.E.-S.A.); farhad.bakhtiary@ukbonn.de (F.B.); 2 Department of Anesthesiology and Intensive Care Medicine, University Hospital Bonn, University of Bonn, Venusberg Campus 1, 53127 Bonn, Germany; markus.velten@ukbonn.de

**Keywords:** aortic surgery, QoL, long-term outcome, SF-36 survey

## Abstract

(1) Background: Despite optimal surgical therapy, replacement of the ascending aorta leads to a significant reduction in the quality of life (QoL). However, an optimal result includes maintaining and improving the QoL. The aim of our study was to evaluate the long-term outcome and the QoL in patients with aneurysms in the ascending aorta; (2) Methods: Between 2014 and 2020, 121 consecutive patients who underwent replacement of the ascending aorta were included in this study. Acute aortic pathologies were excluded. A standard short form (SF)-36 questionnaire was sent to the 112 survivors. According to the surgical procedure, patients were divided into two groups (A: supracoronary replacement of the aorta, n = 35 and B: Wheat-, David- or Bentall-procedures, n = 86). The QoL was compared within these groups and to the normal population, including myocardial infarction (MI), coronary artery disease (CAD) and cancer (CAN) patients; (3) Results: 83 patients were males (68.6%) with a mean age of 62.0 ± 12.5 years. Early postoperative outcomes showed comparable results between groups A and B, with a higher re-thoracotomy rate in B (A: 0.0% vs. B: 22.1%, *p* = 0.002). The 30-day mortality was zero. Overall, mortality during the follow-up was 7.4%. The SF-36 showed a significant decay in both the Physical (PCS) and Mental Component Summary (MCS) in comparison to the normal population (PCS: 41.1 vs. 48.4, *p* < 0.001; MCS: 42.1 vs. 50.9, *p* < 0.001) but without significant difference between both groups. Compared to the MI and CAD patients, significantly higher PCS but lower MCS scores were detected (*p* < 0.05); (4) Conclusions: Replacement of the ascending aorta shows low risk regarding the operative and postoperative outcomes with satisfying long-term results in the QoL. The extent of the surgical procedure does not influence the postoperative QoL.

## 1. Introduction

Aortic aneurysms can affect the entire aorta. Therefore, the treatment of aneurysms in the ascending aorta is only a partial solution of the problem since further progression can occur in other parts of the aorta. Aortic aneurysms could result in acute type A aortic dissection or rupture when left untreated [1,2]. The guidelines recommend a replacement of the AA beyond a diameter of 5.5 cm, unless there are no risk factors, such as the presence of a bicuspid aortic valve or connective tissue diseases [3,4]. Surgery on the thoracic aorta remains challenging and is historically associated with significant mortality and morbidity but is a more standard and safe procedure in centers of excellence [5,6,7,8,9,10,11,12,13]. Despite optimal surgical therapy with significantly lower peri- and postoperative complications, surgery on the aorta could lead to a significant reduction in the quality of life (QoL) [13]. One of the goals in health care is to increase or preserve the QoL of the patient. The optimal result of surgical therapy also includes maintaining and improving the QoL. Especially in asymptomatic and young patients, QoL plays an important role when the surgical success is captured. Although several studies have compared short- and long-term postoperative surgical outcomes, such survival and complications, there are only a few studies in the literature that compares the postoperative QoL of the physical and psychological status after replacement of the AA [8,11,12,13]. Most available studies reported the outcome after acute type A dissection [14,15,16,17]. Some other studies have been performed after interventions of the descending and thoracoabdominal aorta [18,19,20,21,22]. Surgeries on the ascending aorta have become more complex, especially when the aortic root is affected, or a replacement of the aortic valve is necessary [8,11,23]. Therefore, it is not yet known whether the outcome and, in particular, the QoL are affected by the extent of the thoracic aortic surgery performed.

To evaluate the current status and to optimize the therapy, we conducted a survey on the postoperative QoL assessed using the Short-Form-36 (SF-36) health questionnaire in patients with treated aortic aneurysms at our hospital. The main objective of this study was not only to compare the results with the normal population and with patients suffering from other systemic diseases but also as a comparison, depending on the extent and complexity of the surgery.

## 2. Materials and Methods

### 2.1. Patient Selection

**Figure 1:**
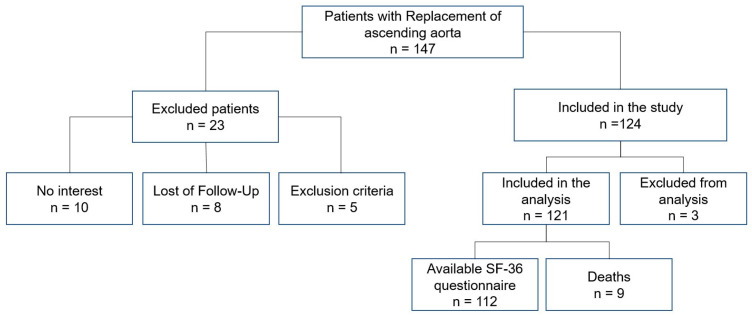
Summary flow diagram of the study population. Abbreviations: SF-36: Short-Form-36 health questionnaire.

Between January 2014 and January 2020, 147 consecutive patients underwent replacement of the ascending aorta due to an aneurysm with or without impairment of the aortic valve and the aortic root at our institution. After approval by the ethics committee of the University of Bonn (No. 180/20) and the written informed consent from all the participating patients, a total of 124 survivors could be traced for this study (Figure 1). Of all the 147 patients, those with involvement of the aortic arch were excluded from this study (n = 5). Three patients had also to been excluded from this study due to a missing SF-36 questionnaire. All patients with emergent aortic repair due to acute type A aortic dissection, aortic rupture or intramural hematoma were also excluded from this study. Therefore, in total 121 could be included in the statistical analysis. According to the surgical procedure, patients were divided into two groups:

A: Supracoronary replacement of the ascending aorta, n = 35;
B: Wheat-, David- or Bentall-procedure; n = 86.

### 2.2. Data Collection

A complete in-hospital data set of pre-, peri- and post-operative parameters were generated and analyzed by the review of patient charts and IT based data sets. The clinical and demographic data and the medical history, including cardiovascular risk factors, former cerebrovascular disease and presenting symptoms, using the NYHA-classification and preoperative risk score systems, such as the European System for Cardiac Operative Risk Evaluation II (EuroScore II), were calculated and analyzed. After telephone contact, the SF-36 questionnaire was sent to the 112 survivors who agreed to participate in this study.

### 2.3. SF-36 Questionnaire

The SF-36 has been found to be reliable and valid for measuring health-related QoL in patients with several chronic disease in several countries worldwide [24]. The questionnaire is a health status profile originally designed to measure patients’ health status and outcomes. The German version of the SF-36 questionnaire was translated by Bullinger in 1995 [25]. It consists of short questions reflecting the QoL using 36 items in eight different aspects:

Physical functioning (PF), Physical Role Functioning (RP), Bodily Pain (BP), General Health (GH), Vitality (VT), Social Functioning (SF), Emotional Role Functioning (RE) and Mental Health (MH). Physical and Mental Component Summary (PCS and MCS) are calculated from the subscales. The higher the score, the better the QoL [26]. The normal German population (n = 6.964) scores, as well as scores from not operated patients with myocardial infarction (MI), coronary artery disease (CAD) and cancer (CAN) were used for analysis [27]. 

### 2.4. Specific Questions

Additional specific questions about current health status in comparison to one year postoperative were included in this survey. The specific questions belonged to four of the eight different aspects of the SF-36 questionnaire subscores: MH, VT, RE and RP. 

### 2.5. Surgical Procedures and Postoperative Course

All surgical procedures were performed via complete median or superior median mini sternotomy. To achieve an activating clotting time of >450 s, anticoagulation with 400 to 500 U/kg sodium heparin was initiated. After cannulation of the ascending aorta and the right atrium, the heart-lung machine was established using a Terumo Advanced Perfusion System 1 (Terumo Cardiovascular Systems, Ann Arbor, MI, USA). The non-heparin coated tube system was primed with crystalloid solution (1000 mL Jonosteril, Fresenius, Bad Homburg, Germany) and 10,000 U of heparin. Patients were routinely cooled to a rectal temperature between 32 and 34 °C, depending on the extent of the procedure. Protection of the myocardium and myocardial arrest were achieved using cardioplegia solution of Brettschneider-HTK (Custodiol, Dr. Franz Kohler Chemie GmbH, Bensheim, Germany). Cardioplegia was either delivered antegrade in the aortic root or directly in the coronary arteries, especially in cases of aortic valve regurgitation. A non-pulsatile pump flow of 2.2 to 2.6 L/min/m^2^ was conducted to maintain a MAP of 50 to 60 mm Hg during the cardiopulmonary bypass (CPB). Commercially available Dacron vascular grafts were used for replacement of the AA. In case of a biological Bentall procedure a Dacron vascular graft and aortic valve prosthesis was sutured together to create the conduit. After careful inspection, the decision on reimplantation or replacement of the aortic valve (David or Bentall-procedure), composite replacement of the ascending aorta and the aortic valve (Wheat-procedure) or an isolated supracoronary replacement depended on the individual morphology as well as on the extent of the aneurysm. The choice between the David procedure or Bentall procedure depended on the morphology of the aortic valve, the patient’s age (younger than 65 years → preference for David) and the surgeons’ experience. All patients were transferred to the ICU postoperatively, where sedation was stopped and an early extubation was aimed for. 

The duration of the surgery, the CPB and the aortic cross-clamping time, the parameters of the organ function and other routine postoperative laboratory variables were recorded. The postoperative outcome data, such as stroke, delirium, myocardial infarction, ventilator-associated pneumonia, the occurrence of pericardial effusion with a need for re-thoracotomy, sepsis, the length of both the ICU and hospital stay as well as 30-day mortality, were recorded.

During the follow-ups, patients were asked for any need for re-hospitalization or a redo of the surgery due to aortic disease or other cardiac disease. The long-term survival was also registered.

### 2.6. Statistical Analysis

The statistical analysis was performed using the IBM SPSS statistics version 25 (SPSS Inc., Chicago, IL, USA) and GraphPad Prism version 8.4.3 (LaJolla). The continuous variables were expressed as mean ± standard deviation (SD) and categorical variables given as absolute values and percentages. The data were tested for a normal distribution using the Kolmogorov–Smirnov and Shapiro–Wilk tests. Normally distributed demographic and clinical patients were analyzed using the students *t*-test. Not normally distributed data were compared using the Mann–Whitney U-test. The categorial variables were evaluated with the Pearson chi-square-test or Fisher’s exact test. The Wilcoxon signed-rank test was used to compare significant differences between each time point. A *p*-value of <0.05 was considered statistically significant. The SF-36 questionnaires were analyzed in accordance with the SF-36 manual. The survival outcomes were assessed by the Kaplan–Meier method and compared using the log-rank test.

## 3. Results

### 3.1. Baseline Characteristics

The baseline characteristics, comorbidities, presenting symptoms, preoperative left ventricular function (EF = ejection fraction) and the EuroScore II are shown in Table 1. Patients were at a mean age of 62.0 ± 12.5 years with 68.6% were male. Patients in group B were significantly younger (A: 67.5 vs. B: 59.8; *p* = 0.003). Significantly more female patients (62.9%) received the isolated supracoronary replacement of the ascending aorta. Patients in group A showed a higher rate of hypertension (A: 91.4% vs. B: 72.1%; *p* = 0.021) and hyperlipidemia (A:63.9% vs. B: 41.9%; *p* = 0.036). No differences in the other recorded comorbidities were found between the two groups. Most of the patients presented with dyspnea according to the NYHA class II-III and with a normal left ventricular function (EF > 50% in more than 85%) without a significant difference between both groups. According to the surgical risk scores, patients were at a low risk for surgery (mean EuroScore II 3.0%) with a significantly higher score in group B (A: 2.69% vs. B: 3.13%; *p* = 0.040). 

### 3.2. Surgical Procedures

Table 2 summarizes the performed surgical procedures. Isolated supracoronary replacement of the AA was performed in 28.9% of patients. Within the group B interventions, the Wheat or Bentall procedures were performed equally. The David procedure at 6.6% of the total collective, was the least frequently performed surgical procedure. In patients with a need for an aortic valve replacement, a biological aortic valve prosthesis was implanted more frequently (83.3% vs. 16.7%).

### 3.3. Postoperative Outcome and Survival 

**Figure 2:**
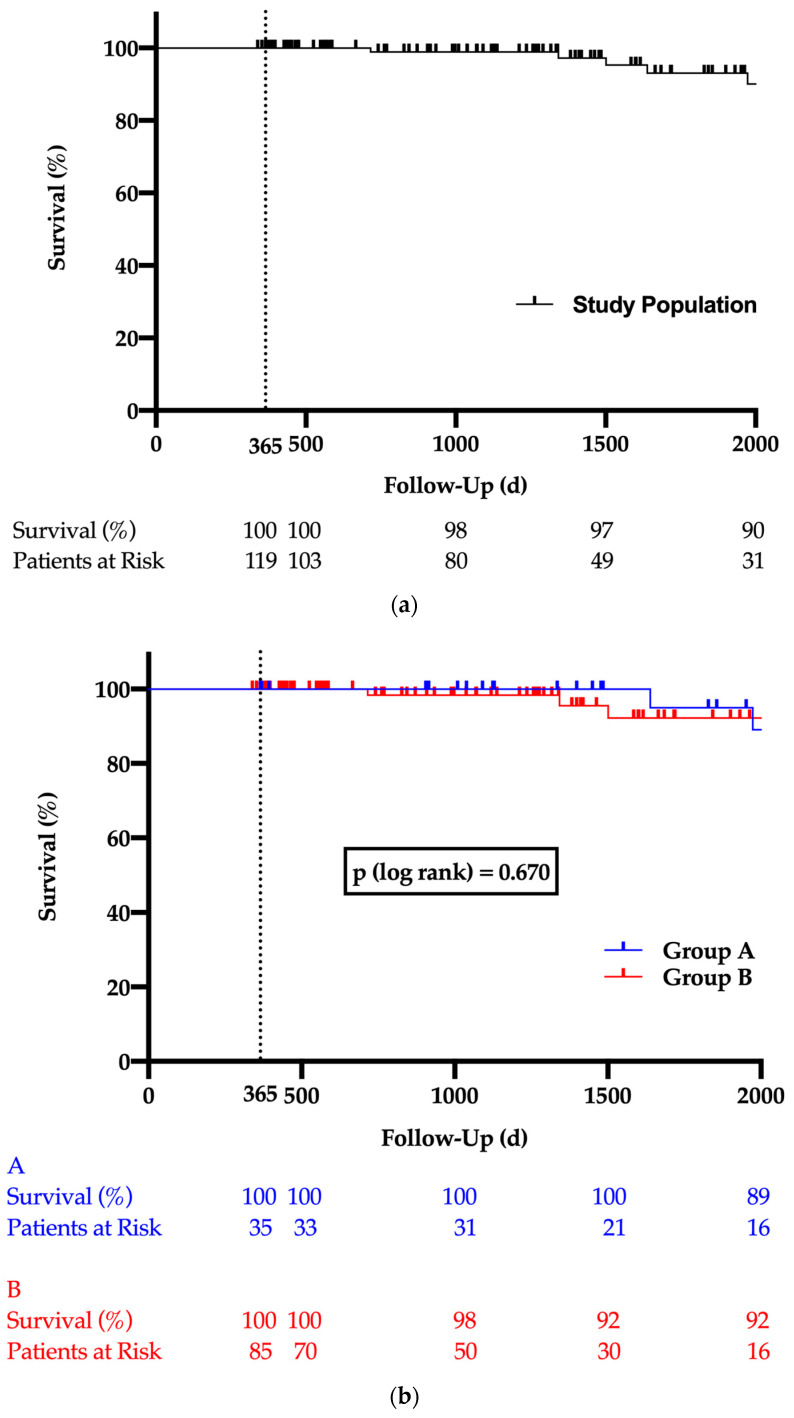
Kaplan–Meyer long-term survival curve for total study population (**a**) and both study groups (**b**). The vertical dashed line highlights the one-year survival.

The postoperative complications are highlighted in Table 3. The mean follow-up time was 46 months with a maximum of 83 months. None of the patients died during the first 30 postoperative days; during the follow-up period, 9 of the 121 patients (7.4%) died as shown in the Kaplan–Meier curve (Figure 2a). The mortality was higher in group A patients during follow-ups but without any statistical significance (A:14.3% vs. B: 4.7%; *p* = 0.119; Figure 2b). Three patients from group B but none from group A experienced peri- or postoperative stroke, also without statistical significance. The delirium rate was 10.7% without any statistical difference between both groups. Group B patients had a significantly higher re-thoracotomy rate (A: 0% vs. B: 22.1%, *p* = 0.002), mostly associated with pericardial effusion (A: 0% vs. B: 10.5%; *p* = 0.058). The mean ICU stay was 3.2 days. Patients were discharged after nearly 15 days, without any statistical differences between the two groups. The other postoperative complications listed in Table 3 are statistically similar between the two groups.

### 3.4. QoL According to SF-36 Questionnaire

**Figure 3:**
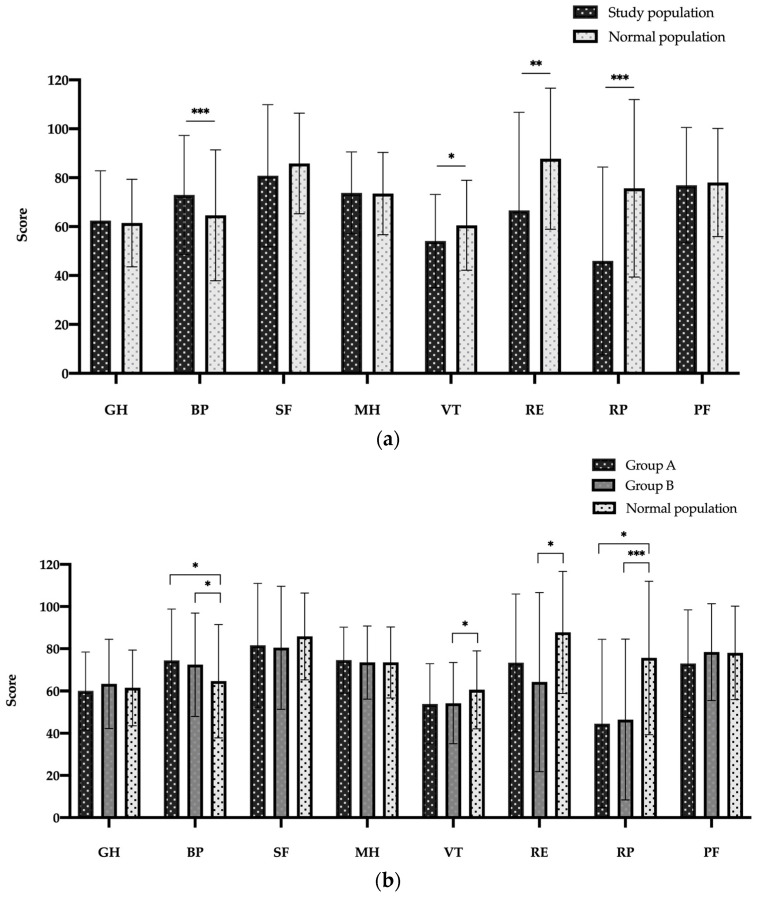
SF-36 scores in eight dimensions for study population (**a**) and for both study groups (**b**) versus normal population. Abbreviations: Bodily Pain (BP), Emotional Role Functioning (RE), General Health (GH), Mental Health (MH), Physical functioning (PF), Physical Role Functioning (RP), Social Functioning (SF), Vitality (VT). Data are expressed as bar charts with mean and ± standard deviation. *p* < 0.05 was considered statistically significant (*). ** *p* < 0.01; *** *p* < 0.001.

Figure 3a,b illustrates all eight SF-36 subscores. Figure 3a shows the comparison between all the patients included in the study with an age-matched and sex-matched German NP value of 1998. The study collective shows differences in the subscores BP, VT, RE and RP. Compared to the normal population, our patients have significantly higher scores in BP, but lower scores in VT, RE and RP. There was no difference in the other SF-36 subscores. Figure 3b shows the different surgical procedures in comparison to the NP. While patients from group B show a difference in the subscores BP, VT, RE and RP compared to the normal population, this difference is only the case for subscores BP and RP in group A patients. There is no difference in any of the eight subscores in the SF-36 questionnaire when comparing patients from groups A and B.

**Figure 4:**
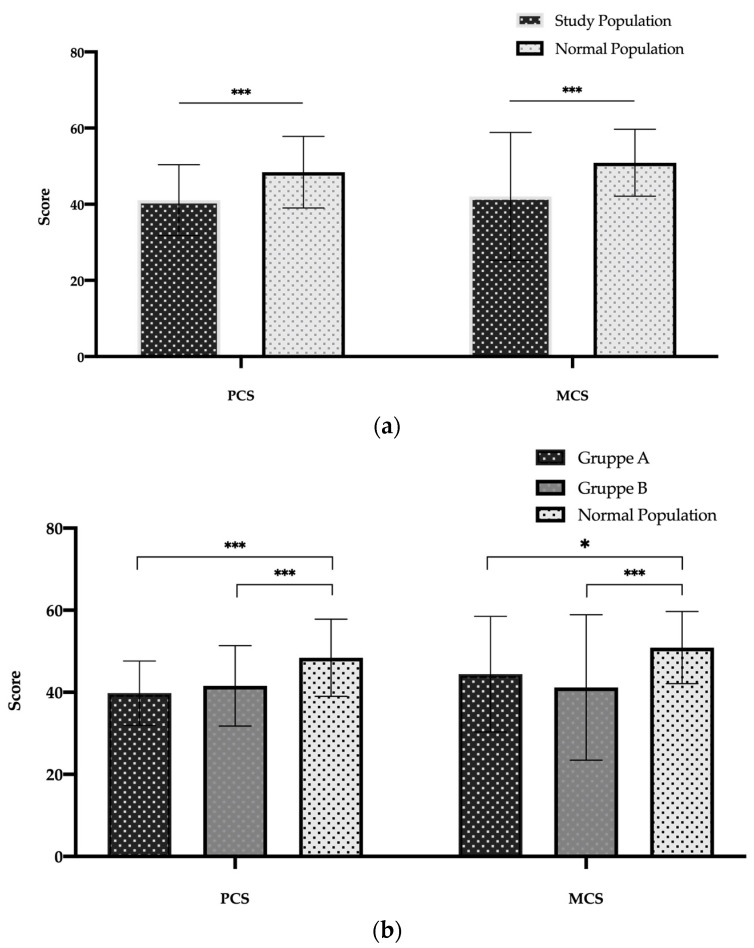
SF-36 Component Summary Scores of study population (**a**) and for both study groups (**b**) versus normal population. Abbreviations: MCS: Mental Component Summary; PCS: Physical Component Summary. Data are expressed as bar charts with mean and ± standard deviation. *p* < 0.05 was considered statistically significant (*). *** *p* < 0.001.

The PCS and MCS measurements of all patients in comparison with the normal population are displayed in Figure 4a,b. The PCS and MCS values of the study population are both significantly lower than the values of the healthy normal population (*p* < 0.001, Figure 4a). Dividing the study population based on the surgical procedure and comparing their PCS and MCS values with values of a normal healthy German population, the same negative trend with significantly lower PCS and MCS values were shown. However, there is no difference in the PCS and MCS values between groups A and B. 

**Figure 5:**
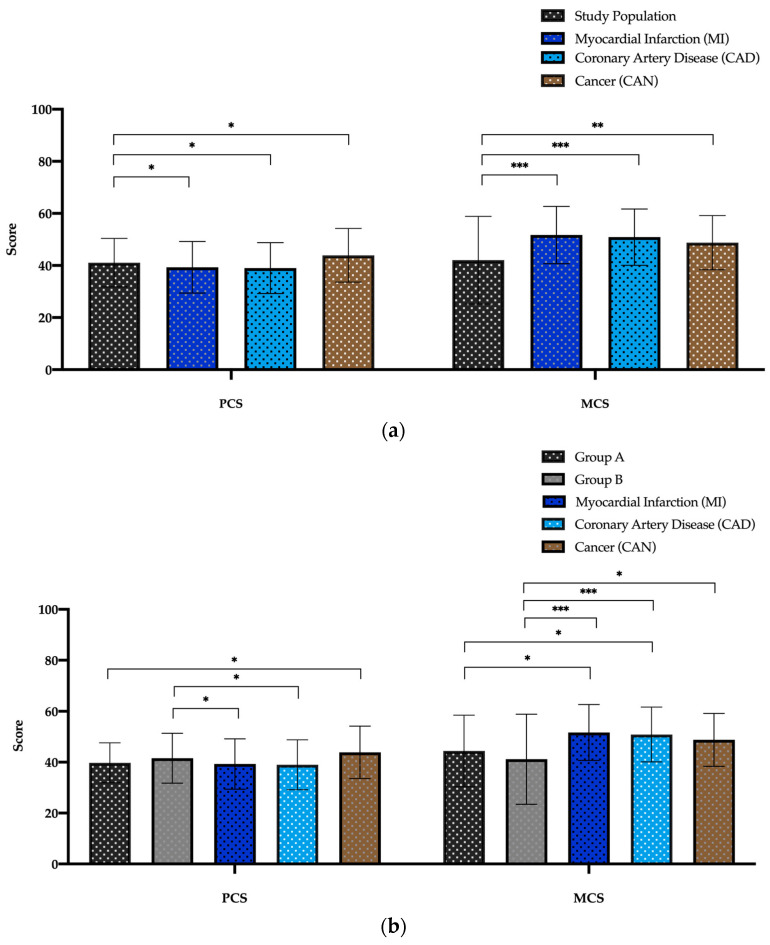
SF-36 Component Summary Scores of study population (**a**) and for both study groups (**b**) versus Myocardial Infarction (MI), Coronary Artery Disease (CAD) and Cancer (CAN) patients. Abbreviations: MCS: Mental Component Summary; PCS: Physical Component Summary. Data are expressed as bar charts with mean and ± standard deviation. *p* < 0.05 was considered statistically significant (*). ** *p* < 0.01; *** *p* < 0.001.

The comparison between the PCS and MCS values of both the study groups with different diseases of the 1998 Federal German Health survey are illustrated in Figure 5a,b. PCS is significantly better in the study population than MI and CAD but worse than CAN patients (Figure 5a). In the MCS measurement a reverse trend is shown when comparing the scores of the study population to MI, CAD and CAN patients (Figure 5b). According to the study groups, patients of group A show no differences in the PCS values compared to MI and CAD, but not to the CAN patients. Group B patients, on the other hand, have better values compared to MI and CAD, but no difference to the CAN patients. In the MCS category, both groups have worse values than MI and CAD but only group B patients have worse values in comparison to the CAN patients (Figure 5b).

For patients from group B, we compared the QoL of the Bentall/Wheat (group C) and David (group D) patients. The results are highlighted in Figure S1a,b (Supplementary Materials). No significant differences were seen between the two groups in the subgroups of the SF-36 questionnaire as well as in the PCS and MCS scores.

### 3.5. Follow-Up Questionnaire

As shown in Table 3, the need for a redo surgery during a follow-up was 1.6%, whereby an aortic re-intervention was very low (0.8%), without any statistical significance between both the study groups. Of all the study patients who were still employed (n = 71), only 81.7% were able to return to work during the follow-up, with no statistical difference between the two study groups (*p* = 0.664).

**Figure 6:**
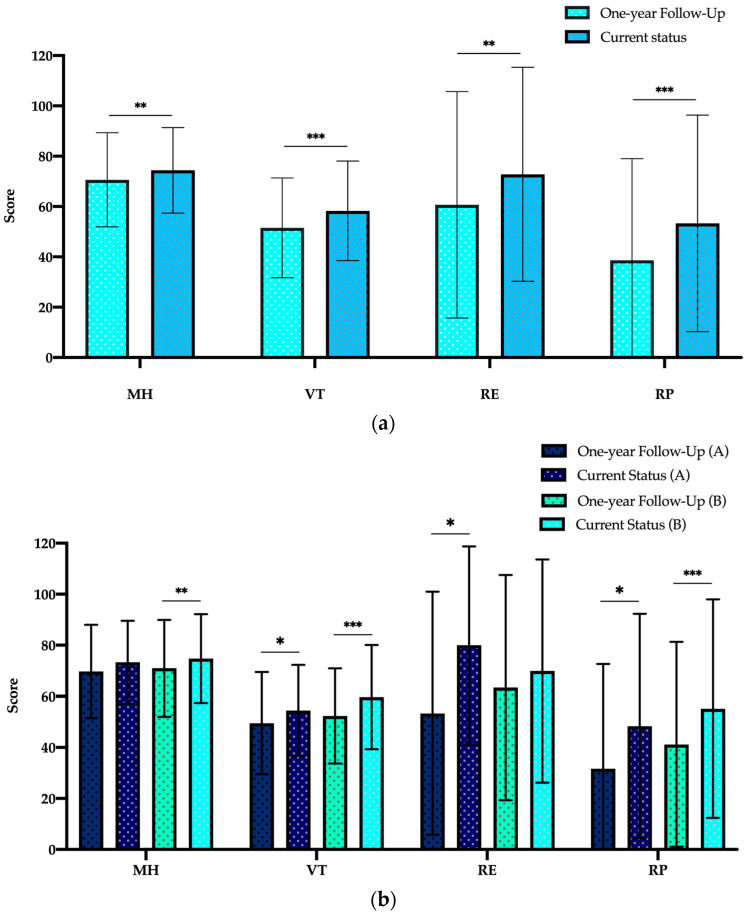
Comparison between one-year follow-up and current status of SF-36 scores in four dimensions for study population (**a**) and for both study groups (**b**). Abbreviations: Emotional Role Functioning (RE), Mental Health (MH), Physical Role Functioning (RP) and Vitality (VT). Data are expressed as bar charts with mean and ± standard deviation. *p* < 0.05 was considered statistically significant (*). ** *p* < 0.01; *** *p* < 0.001.

During the follow-ups, patients were asked in four subscores of the SF-36 questionnaire (MH, VT, RE and RP) to compare their QoL one year after surgery. As shown in Figure 6a,b, in all four categories the health status improved significantly during FU (Figure 6a). Group A patients show this improvement in VT, RE and RP and group B patients in MH, VT and RP (Figure 6b).

## 4. Discussion

Surgery of the ascending aorta remains a challenge. Despite improvement in surgical procedures and very low postoperative mortality and morbidity, patients experience a significant reduction in their QoL during follow-ups, as most of the patients are asymptomatic at the time of surgery. The optimal therapeutic outcome includes, not only a successful surgery, but the maintenance or even improvement in patients’ QoL. The aim of our study was to evaluate patients’ QoL using the SF-36 questionnaire after the isolated replacement of the ascending aorta with or without involvement of the aortic valve. In this retrospective observational study using follow-up questions on their QoL, we compared the QoL of the patients operated upon with that of a normal healthy German population. In addition, the QoL was compared to three selected common diseases in Europe MI, CAD, and CAN. Furthermore, the extent of the surgical procedure on the QoL was investigated. We were able to show that similar results were obtained in many subgroups of the SF-36 questionnaire in both the short- and long-term follow-up of our study population compared with the normal population. Compared to the short-term follow-up (one year after surgery), the QoL improved significantly in the long-term follow-up. Our patients scored worse values in PCS and MCS compared with the normal population. However, our study population was better in PCS when compared to MI and CAD patients, but not in the MCS category. Compared with CAN patients, the study population scored worse in PCS and MCS, respectively. The extent of the surgical procedure had no influence on the QoL. Patients who received only replacement of the ascending aorta were mostly female and eight years older than patients with the additional need of reconstruction or replacement of the aortic valve. In the long-term follow-up, we could show a survival rate of approximately 91%, with a maximum observation period of 83 months. The re-intervention rate was less than 2% in the long-term follow-up. 

Recently, there has been a significant increase in the importance of health related QoL, including the physical, psychological and social aspects of life affected by a disease process and its treatment. The current literature includes few studies that reflect the QoL of patients in the long-term follow-up after the isolated replacement of the ascending aorta in aneurysm patients. The majority of the existing studies confirm acceptable outcomes for the QoL after major thoracic aortic surgery compared with the normal population. QoL is an important cornerstone for a satisfied postsurgical outcome. QoL questionnaires are now used more extensively. Surgical therapy can be perfect but if the patients do not feel an improvement in their QoL after the surgery, the overall result is not satisfactory. 

In 1999, Olsson et al. presented in a retrospective study their results about the QoL in patients undergoing surgery of the thoracic aorta for all types of aortopathy in 81 patients. Their patients were of a mean age of 59 years with 70% being male. They also used the SF-36 questionnaire to measure the QoL. Similar to our results, their patients showed an improved or preserved QoL after surgery. Ninety-one percent of the patients considered the operation successful. Overall, the QoL in this population, measuring the PCS and MCS, was significantly lower than in a matched normal population, especially in the RE and RP subscales but not in the BP [13]. We also recorded lower PCS and MCS values in comparison to the normal population. 

The impact of the different aortic surgical procedures on outcome and QoL was published by Stadler et al. in 2007 in a cohort of 244 patients with a mean age of 60 years and 75% of male gender. One third of their patients had an acute type A aortic dissection. Nearly 52% of the patients received a Bentall-procedure and 17% and 31% underwent a Wheat-procedure or an isolated supracoronary replacement of the aorta, respectively. In a follow-up period of 26 months, they showed that the QoL was comparable to an age- and sex-matched population after treatment of the thoracic aorta, including the aortic root. Overall, the in-hospital mortality rate was 6.1% and 8.1% of the patients suffered a neurological event [8]. Although stroke and in-hospital mortality rates were lower in our study population, in contrast to Stadler et al. we excluded all dissection patients, which could be an explanation for the lower results in our study. 

Franke et al. compared the QoL of 143 patients who underwent the David-procedure or mechanical Bentall-procedure with a mean age of 58 years. Regarding the early postoperative outcome, a stroke occurred significantly more in the Bentall group both in-hospital and during follow-ups. A five-year survival of nearly 85% was equal between the two groups and comparable with our results. Additionally, using the SF-36 survey, the QoL was found to be significantly impaired in all the SF-36 subscales, apart from BP and SF, for patients belonging to the Bentall group when compared with the David group [11]. Bentall patients significantly reported to feeling moderately or severely disturbed by the noise of the prosthetic valve. In a sub-analysis, we compared the results of the SF-36 questionnaire between the David- and Bentall/Wheat-procedure without any significant difference. However, a statistical comparison is difficult in such low numbers of patients in the David group (n = 8). 

Early and mid-term result analyses in patients undergoing aortic root replacement, using the Bentall-procedure, were published by Lehr et al. in 2011. The authors compared the outcome between biological and mechanical valves. One- and five-year survival rates for the mechanical group were 96.0% and 89.0%, respectively, vs. 93.0 and 84.0% for the biological group. The valve type was not predictive of all-cause mortality or valve-related complications. Using the EQ-5D (Euroqol) questionnaire they showed that general and disease-specific health-related QoL scores were not significantly different between the two study groups [23]. In the Bentall group of our present study, the biological conduit was chosen most frequently. Nowadays, with the possibility of TAVI procedure in the follow-up, we will probably rarely see mechanical conduits in the future. This could be an advantage, especially for younger patients, as they often feel disturbed by the valve noise, which could influence their QoL. 

In 2015, Endlich et al. presented data about the long-term outcome and QoL of acute type A aortic dissection patients. In their study they included 120 patients with a mean age of nearly 60 years. During an observational time of a maximum of 156 months, the overall mortality was 39%. The SF-36 observation showed a significant decay in both PCS and MCS, without improvement during the follow-up, especially in the younger patients. Endlich et al. also compared PCS and MCS of acute type A aortic dissection patients with MI, CAD and CAN patients. Similar to our results, they recorded significantly better PCS values than MI and CAD patients, but worse MCS values. Comparing the PCS and MCS to CAN patients, they did not show any differences [15].

Currently, three other studies were presented showing the importance of surveying the QoL after aortic surgery [28,29,30]. Barrena-Blázquez et al. examined, in a cross-sectional study, the association of age and surgical technique with the QoL of 151 male patients who received treatment for an aneurysm in the abdominal aorta. They could show a significant decrease in the SF, VT, MH and PF scores using the SF-36 questionnaire in comparison to older patients, and the type of performed surgical technique on the PF and MH scores [28]. In our study we did not examine the association of age on the QoL but we could show a decrease in the VT score; the extent of the surgical technique did not influence the QoL outcome in our patients. 

The second study was a large prospective study in the UK with more than 800 patients with untreated thoracic aneurysms in 30 vascular/cardiothoracic units published in 2022 by Sharples et al. The aim of this study was to observe, describe and evaluate the management and timing of intervention for patients suffering from untreated aortic aneurysms. Although aneurysm size did not play a role in the decrease in the QoL, the authors could show a significant annual decrease in the QoL for each ten year increase in age [29]. 

Third, in 2021 Smolock et al. assessed and compared the QoL in 146 patients after combined proximal and distal aortic replacement using open or multimodal/endovascular approaches. After a median follow-up of 6.2 years, they identified a greater number of postoperative complications and a history of COPD as predictors for a worse postoperative QoL compared to the normal population. The surgical approach did not influence the QoL; therefore, they concluded that the recommendation of the surgical approach should be chosen by the surgeon’s experience [30]. This study also shows how important it is to survey the QoL of patients. Although patients are treated, the knowledge that aortic re-interventions could be required causes a significant reduction in the QoL. 

Regarding all these presented studies, a true comparison to our study is limited due to the different diseases included in the study population. Most of the studies included acute type A aortic dissection patients, which were treated emergently. Our patients had been presented for elective surgery. All cases of emergent surgeries were excluded from the current study. Most of the prior studies did not compare the QoL of patients who received an isolated replacement of the ascending aorta with patients were the aortic valve needed an intervention. To date, the extent and impact of the surgical therapy on the QoL has not been studied in detail in former studies. Also, reliable long-term data are also lacking. 

A study that is almost comparable to ours is the study by Lohse et al., which was published in 2009 and presented the QoL assessments for 134 consecutive patients with true aneurysms undergoing AA surgery. Lohse et al. included only patients who underwent nonemergent aortic repair and excluded all patients with emergent aortic repair due to acute type A aortic dissection, aortic rupture or intramural hematoma. The mean age was 61 years, similar to our patients. The mean follow-up period was 36 months. The thirty-day and mid-term mortality rates were 3.7 and 3.9%, respectively. The rates of stroke and bleeding were 6% and of myocardial infarction 4.4% [12]. The bleeding rate was 15.7%, which was higher in our study, mainly occurring in group B with complex surgical procedures, which can explain the higher re-thoracotomy rate. In contrast to our study, Lohse et al. did not compare the bleeding results depending on the surgical procedure. Regarding the QoL, it revealed that physical function results were significantly lower than the reference population in patients within range of 70–79 years of age. In most other scores, there was no significant difference when the QoL was compared with the reference age- and sex-matched population. The authors also used the SF-36 questionnaire to measure the QoL and concluded that this invasive procedure can be performed with good mid-term results and an acceptable QoL without further differentiation [12].

All the studies presented, including the results from our study, show the importance of surveying the QoL after thoracic aortic procedures. The results of surgery on the ascending aorta are satisfying. Procedures on the ascending aorta can be performed with a high degree of safety. However, the constant concern that complications or that other aortic pathologies may develop downstream of the aorta, as well as an ongoing need for follow-ups and the use of medications, provides disease awareness and the feeling of imminent danger, especially in younger patients. This seems to have a significant impact on the mental components of the QoL, which is more serious in relation to the physical components. The fact that almost one fifth of the employed patients lost their jobs or had to retire shows, especially for the younger patients, that there is a need for psychosomatic support throughout the course of treatment to be able to overcome this severe disease well postoperatively. The fact that CAN patients had better scores in MCS than our patients, seems surprising at first. However, considering that CAN patients often receive psychological support, these better results can be explained; therefore, this should also be the case for patients who have undergone surgery on the thoracic aorta too. 

## 5. Conclusions

Surgery on the thoracic aorta remains challenging and is associated with an impairment of the QoL during follow-ups compared to a normal healthy population. Replacement of the ascending aorta shows a low risk regarding operative and postoperative outcomes with excellent long-term results. In most subscores of the SF-36 questionnaire, which is the most popular and recommended instrument to be used, the QoL is similar to the normal population. The PCS, but not MCS values, are even better than the values of the MI or CAD patients. However, the mental component is more serious in relation to PCS, especially in younger asymptomatic patients. In these patients, psychological therapy may improve the mental component of the QoL in the follow-up. The extent of the surgical procedure does not influence the postoperative QoL. More data collection in randomized studies with a baseline QoL and a follow-up QoL are needed.

## 6. Limitation

First, the study was limited by its retrospective nature, suggesting the possibility of selection bias or that the two patient groups may not have been truly comparative. Second, the study was conducted at a single center with a low sample size, which may affect the generalizability of the findings in a broader population. Third, there was no preoperative QoL evaluation due to the retrospective character of the study; therefore, a baseline QoL is missing and could not be compared with the postoperative QoL.

## Figures and Tables

**Table 1:** Patient preoperative baseline characteristics and preexisting conditions.

	Total (n = 121)	Group A (n = 35)	Group B (n = 86)	*p*-Value
Age	62.02 ± 12.54	67.51 ± 9.13	59.78 ± 13.08	*0.003*
Sex				<*0.001*
male	83 (68.6%)	13 (37.1%)	70 (81.4%)	
female	38 (31.4%)	22 (62.9%)	16 (18.6%)	
BMI	27.67± 5.23	28.91 ± 5.18	27.17 ± 5.20	0.107
Comorbidities				
Hypertension	94 (77.7%)	32 (91.4%)	62 (72.1%)	*0.021*
Hyperlipidemia	58 (47.9%)	22 (63.9%)	36 (41.9%)	*0.036*
Diabetes mellitus	11 (9.1%)	5 (14.3%)	6 (7.0%)	0.293
COPD	15 (12.4%)	3 (8.6%)	12 (14.0%)	0.550
Smoker	52 (43.0%)	16 (45.7%)	36 (41.9%)	0.698
pAVD	4 (3.3%)	0 (0.0%)	4 (4.7%)	0.322
cAVD	6 (5.0%)	3 (8.6%)	3 (3.5%)	0.354
Stroke	12 (9.9%)	3 (8.6%)	9 (10.5%)	0.752
Atrial fibrillation (AF)	38 (31.4%)	12 (34.3%)	26 (30.2%)	0.663
Coronary heart disease (CAD)	28 (23.1%)	6 (17.1%)	22 (25.6%)	0.318
Myocardial infarction (MI)	5 (4.1%)	1 (2.9%)	4 (4.7%)	1.000
Former PCI/Stenting	3 (2.5%)	1 (2.9%)	2 (2.3%)	0.530
Chronic renal failure	6 (5.0%)	0 (0.0%)	6 (7.0%)	0.180
Ejection fraction				0.532
>50%	104 (86.0%)	31 (88.6%)	73 (84.9%)	
30–50%	14 (11.6%)	4 (11.4%)	10 (11.6%)	
<30%	3 (2.5%)	0 (0.0%)	3 (3.5%)	
NYHA classification				0.313
0	11 (9.1%)	4 (11.4%)	7 (8.1%)	
I	31 (25.6%)	10 (28.6%)	21 (24.4%)	
II	38 (31.4%)	11 (31.4%)	27 (31.4%)	
III	40 (33.1%)	10 (28.6%)	30 (34.9%)	
IV	1 (0.8%)	0 (0.0%)	1 (1.2%)	
Former Surgeries				
Cardiac surgery	8 (6.6.%)	4 (11.4%)	4 (4.7%)	0.227
Aortic surgery	5 (4.1%)	1 (2.9%)	4 (4.7%)	1.000
Scores				
Euroscore II	3.00 ± 1.57	2.69 ± 1.64	3.13 ± 1.53	*0.040*

Abbreviations: BMI: Body Mass Index; cAVD: cerebral Arterial Vascular Disease; COPD: Chronic Obstructive Pulmonary Disease; EuroScore II: European System for Cardiac Operative Risk Evaluation II; NYHA: New York Heart Association; pAVD: peripheral Arterial Vascular Disease; PCI: Percutaneous Coronary Intervention. Notes: Group A: Supracoronary replacement of the ascending aorta, Group B: Wheat-, Bentall- or David-procedure. Values are expressed as mean ± standard deviation or as number and percentage (in bracket). Significant results are displayed in italics.

**Table 2:** Procedural details.

Surgical Procedure	Patients n = 121
Supracoronary replacement of AA	35 (28.9%)
Wheat	40 (33.1%)
Bentall	38 (31.4%)
David	8 (6.6%)
Aortic Valve Prosthesis	
biological	65/78 (83.3%)
mechanical	13/78 (16.7%)

**Table 3:** Postoperative Outcome and Follow-up details.

	Total (n = 121)	Group A (n = 35)	Group B (n = 86)	*p*-Value
Postoperative Complication				
Stroke	3 (2.5%)	0 (0.0%)	3 (3.5%)	0.556
Delirium	13 (10.7%)	4 (11.4%)	9 (10.5%)	1.000
Pericardial effusion	9 (7.4%)	0 (0.0%)	9 (10.5%)	0.058
Re-thoracotomy due to bleeding	19 (15.7%)	0 (0.0%)	19 (22.1%)	*0.002*
Myocardial infarction	1 (0.8%)	0 (0.0%)	1 (1.2%)	1.000
Sepsis	3 (2.5%)	1 (2.9%)	2 (2.3%)	1.000
Pneumonia	7 (5.8%)	2 (5.7%)	5 (5.8%)	1.000
Respiratory failure	16 (13.2%)	2 (5.7%)	14 (16.3%)	0.148
Wound infection	6 (5.0%)	0 (0.0%)	6 (7.0%)	0.180
30-day mortality	0 (0.0%)	0 (0.0%)	0 (0.0%)	-
In hospital stay	14.57 ± 6.78	12.43 ± 3.38	15.44 ± 7.60	0.124
ICU stay	3.22 ± 4.63	2.14 ± 1.63	3.66 ± 5.34	0.125
Discharge				0.421
home	113 (93.4%)	34 (97.1%)	79 (91.9%)	
rehabiltation	4 (3.3%)	0 (0.0%)	4 (4.7%)	
Other acute care hospital	4 (3.3%)	1 (2.9%)	3 (3.5%)	
Follow-up				
death	9 (7.4%)	5 (14.3%)	4 (4.7%)	0.119
re-operation during follow-up	2 (1.6%)	0 (0.0%)	2 (1.2%)	1.000
Employment status	71 (58.7%)	13 (37.1%)	58 (67.4%)	0.667
retired	14 (19.7%)	2 (15.4%)	12 (20.7%)	
returned to work	57 (80.3%)	11 (84.6%)	46 (79.3%)	

Abbreviations: ICU: Intensive Care Unit. Notes: Group A: Supracoronary replacement of the ascending aorta, Group B: Wheat-, Bentall- or David-procedure. Values are expressed as mean ± standard deviation, or as number and percentage (in bracket). Significant results are displayed in italics.

## Data Availability

The data presented in this study are available on request from the corresponding author.

## Supplementary Materials

The following supporting information can be downloaded at: https://www.mdpi.com/article/10.3390/jcm12134498/s1, Figure S1: SF-36 scores in eight dimensions (a) and Component Summary Scores (b) for Bentall/Wheat- (Group C) versus David-procedure (Group D).
