# Supplementary material for: The Long-Term Outcome and Quality of Life after Replacement of the Ascending Aorta
Source: J Clin Med. 2023 Jul 5;12(13):4498. doi: 10.3390/jcm12134498 (PMC10342701; doi:10.3390/jcm12134498)
Supplement: Supplementary file 1 [file jcm-12-04498-s001.zip › jcm-2434239-supplementary.pdf]

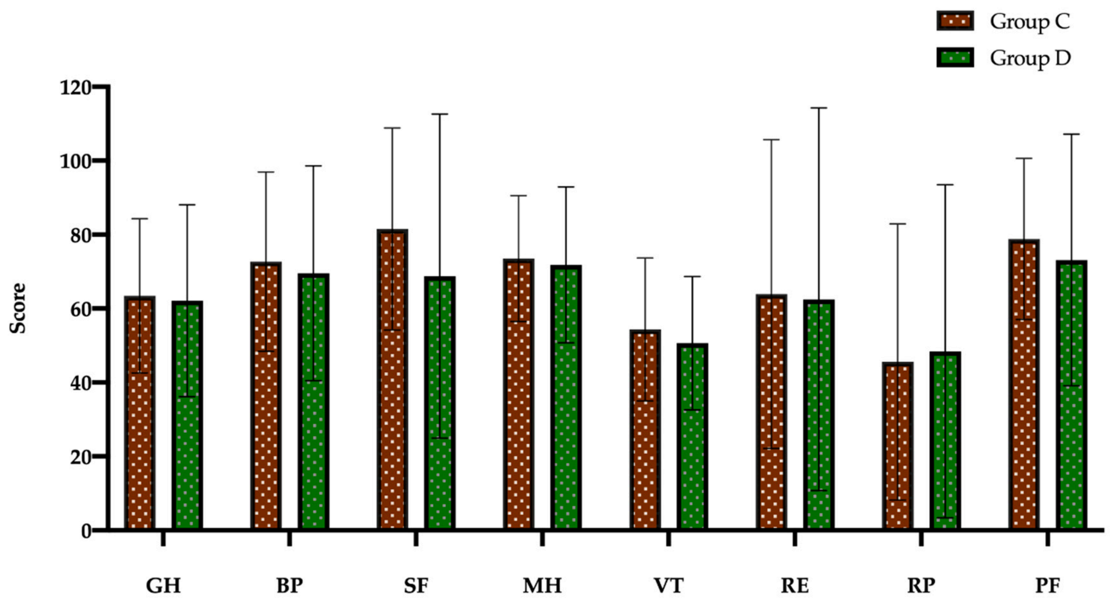

(a)

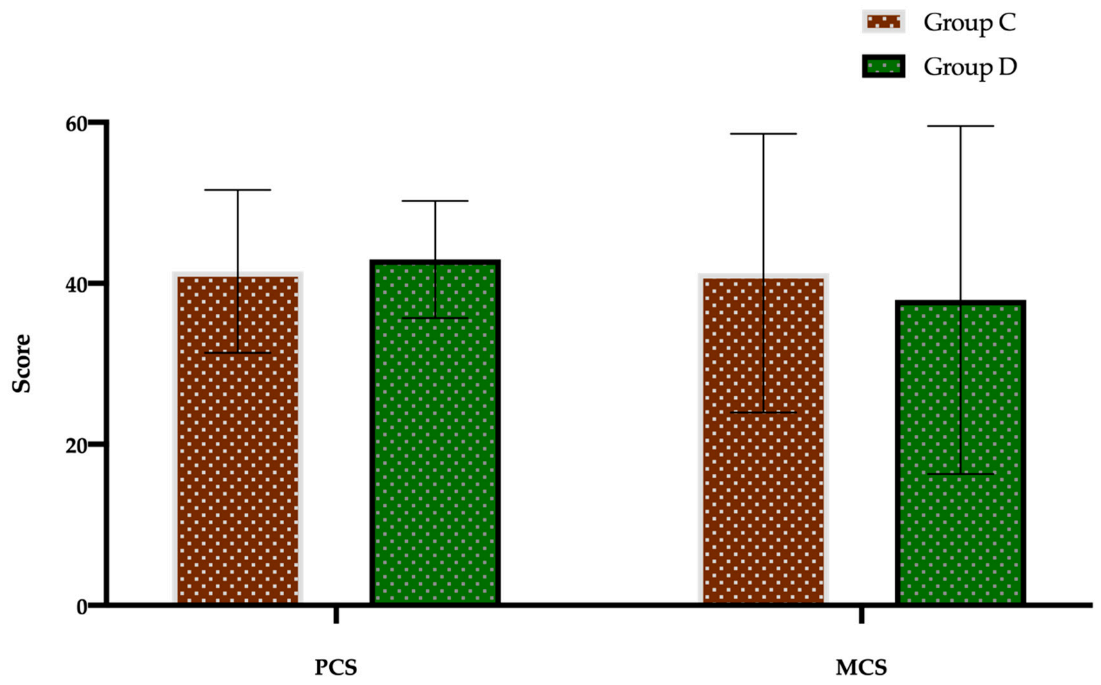

(b)

Figure S1a-b. SF-36 scores in eight dimensions **(a)** and Component Summary Scores **(b)** for Bentall/Wheat- (Group C) versus David-procedure (Group D). Abbreviations: Bodily Pain (BP), Emotional Role Functioning (RE), General Health (GH), Mental Health (MH), MCS: Mental Component Summary, PCS: Physical Component Summary, Physical functioning (PF), Physical Role Functioning (RP), Social Functioning (SF), Vitality (VT). Data are expressed as bar charts with mean and ± standard deviation.
